# Diagnosis and management of migraine in ten steps

**DOI:** 10.1038/s41582-021-00509-5

**Published:** 2021-06-18

**Authors:** Anna K. Eigenbrodt, Håkan Ashina, Sabrina Khan, Hans-Christoph Diener, Dimos D. Mitsikostas, Alexandra J. Sinclair, Patricia Pozo-Rosich, Paolo Martelletti, Anne Ducros, Michel Lantéri-Minet, Mark Braschinsky, Margarita Sanchez del Rio, Oved Daniel, Aynur Özge, Ayten Mammadbayli, Mihails Arons, Kirill Skorobogatykh, Vladimir Romanenko, Gisela M. Terwindt, Koen Paemeleire, Simona Sacco, Uwe Reuter, Christian Lampl, Henrik W. Schytz, Zaza Katsarava, Timothy J. Steiner, Messoud Ashina

**Affiliations:** 1 Danish Headache Center, Department of Neurology, Rigshospitalet Glostrup, Faculty of Health and Medical Sciences, University of Copenhagen, Copenhagen, Denmark; 2 Institute for Medical Informatics, Biometry and Epidemiology, Medical Faculty, University Duisburg-Essen, Essen, Germany; 3 First Department of Neurology, Aeginition Hospital, National and Kapodistrian University of Athens, Athens, Greece; 4 Metabolic Neurology, Institute of Metabolism and Systems Research, College of Medical and Dental Sciences, University of Birmingham, Birmingham, UK; 5 Birmingham Neuro-Ophthalmology, Queen Elizabeth Hospital, Birmingham, UK; 6 Centre for Endocrinology, Diabetes and Metabolism, Birmingham Health Partners, Birmingham, UK; 7 Department of Neurology, University Hospitals Birmingham NHS Foundation Trust, Queen Elizabeth Hospital, Birmingham, UK; 8 Headache Unit, Neurology Department, Vall d’Hebron University Hospital, Barcelona, Spain; 9 Headache and Neurological Pain Research Group, Vall d’Hebron Research Institute, Universitat Autònoma de Barcelona, Barcelona, Spain; 10 Department of Clinical and Molecular Medicine, Sapienza University, Rome, Italy; 11 Regional Referral Headache Centre, Sant’Andrea Hospital, Rome, Italy; 12 Neurology Department, Montpellier University Hospital, Montpellier, France; 13 Departement d’Evaluation et Traitement de la Douleur, Centre Hospitalo-Universitaire de Nice, Nice, France; 14 Neurology Clinic, Tartu University Hospital, Tartu, Estonia; 15 Department of Neurology, Clínica Universidad de Navarra, Madrid, Spain; 16 Headache & Facial Pain Clinic, Laniado Medical Center, Netanya, Israel; 17 Department of Neurology, Mersin University Medical Faculty, Mersin, Turkey; 18 Department of Neurology, Azerbaijan State Medical University, Baku, Azerbaijan; 19 Department of Anesthesiology and Intensive Care, P. Stradins University, Riga, Latvia; 20 University Headache Clinic, Moscow, Russia; 21 Ukrainian Institute of Family Medicine, Kiev, Ukraine; 22 Department of Neurology, Leiden University Medical Center, Leiden, Netherlands; 23 Department of Neurology, Ghent University Hospital, Ghent, Belgium; 24 Neuroscience Section, Department of Applied Clinical Sciences and Biotechnology, University of L’Aquila, L’Aquila, Italy; 25 Department of Neurology, Charité Universitätsmedizin Berlin, Berlin, Germany; 26 Headache Medical Center, Seilerstaette Linz, Linz, Austria; 27 Department of Geriatric Medicine, Ordensklinikum Linz, Linz, Austria; 28 Department of Neurology, University of Duisburg-Essen, Essen, Germany; 29 Department of Neurology, Evangelical Hospital Unna, Unna, Germany; 30 EVEX Medical Corporation, Tbilisi, Georgia; 31 Department of Nervous Diseases of the Institute of Professional Education, IM Sechenov First Moscow State Medical University, Moscow, Russia; 32 Department of Neuromedicine and Movement Science, Faculty of Medicine and Health Sciences, NTNU Norwegian University of Science and Technology, Trondheim, Norway; 33 Division of Brain Sciences, Imperial College London, London, UK; 34 Danish Knowledge Center on Headache Disorders, Glostrup, Denmark; 35 Department of Neurology, Azerbaijan Medical University, Baku, Azerbaijan

**Keywords:** Migraine, Migraine

## Abstract

Migraine is a disabling primary headache disorder that directly affects more than one billion people worldwide. Despite its widespread prevalence, migraine remains under-diagnosed and under-treated. To support clinical decision-making, we convened a European panel of experts to develop a ten-step approach to the diagnosis and management of migraine. Each step was established by expert consensus and supported by a review of current literature, and the Consensus Statement is endorsed by the European Headache Federation and the European Academy of Neurology. In this Consensus Statement, we introduce typical clinical features, diagnostic criteria and differential diagnoses of migraine. We then emphasize the value of patient centricity and patient education to ensure treatment adherence and satisfaction with care provision. Further, we outline best practices for acute and preventive treatment of migraine in various patient populations, including adults, children and adolescents, pregnant and breastfeeding women, and older people. In addition, we provide recommendations for evaluating treatment response and managing treatment failure. Lastly, we discuss the management of complications and comorbidities as well as the importance of planning long-term follow-up.

## Abstract (web summary)

In this Consensus Statement, which is endorsed by the European Headache Federation and the European Academy of Neurology, an expert panel provides recommendations for the diagnosis and management of migraine to support clinical decision-making by general practitioners, neurologists and headache specialists.

## Introduction

Migraine is a highly disabling primary headache disorder with a 1-year prevalence of ~15% in the general population^1,2^. According to the Global Burden of Disease Study, migraine is the second most prevalent neurological disorder worldwide and is responsible for more disability than all other neurological disorders combined^2,3^.

Migraine manifests clinically as recurrent attacks of headache with a range of accompanying symptoms^4^. In approximately one third of individuals with migraine, headache is sometimes or always preceded or accompanied by transient neurological disturbances, referred to as migraine aura^5,6^. Furthermore, a minority of those affected develop chronic migraine, in which attacks become highly frequent^7^. The pathogenesis of migraine is widely believed to involve peripheral and central activation of the trigeminovascular system^8^, and cortical spreading depression is thought to be the underlying neurophysiological substrate of migraine aura^9^. However, much remains unknown about specific pathogenic processes and few mechanism-based treatment options currently exist^10^.

Treatments for migraine include acute and preventive medications and a range of non-pharmacological therapies^10^. Despite these treatment options and the comprehensive diagnostic criteria, clinical care remains suboptimal — misdiagnosis and under-treatment of migraine are substantial public health challenges^11,12^. Population-based data from Europe indicate that preventive medication for migraine is used by only 2–14% of eligible individuals^11^, an alarming finding that calls for global action^12^. A comprehensive approach is needed to facilitate accurate diagnosis and evidence-based management.

**Fig. 1:**
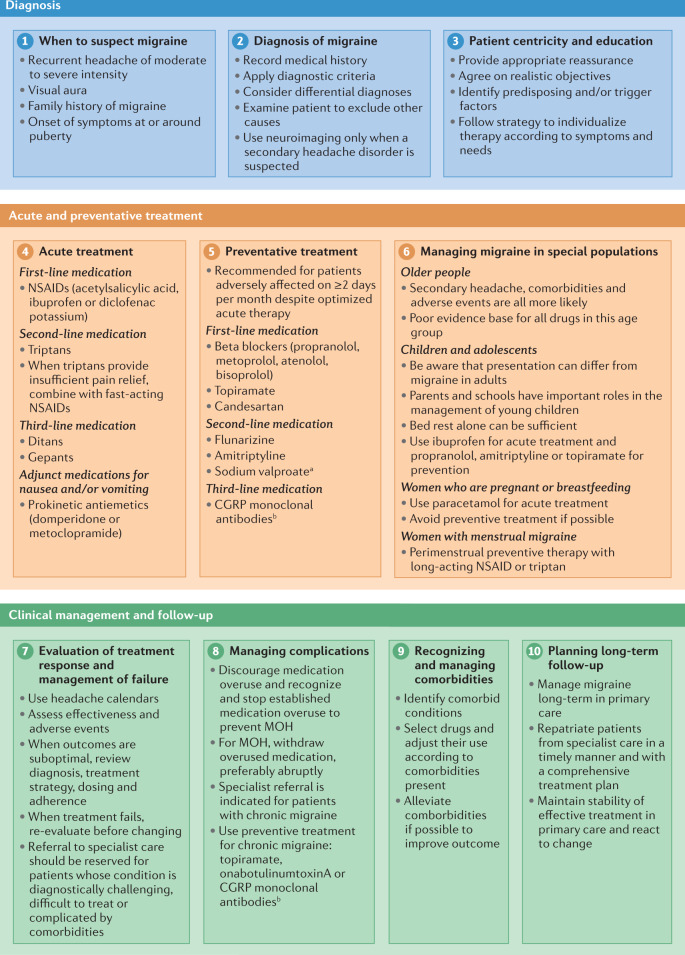
Ten-step approach to the diagnosis and management of migraine. CGRP, calcitonin gene-related peptide; MOH, medication overuse headache; NSAID, non-steroidal anti-inflammatory drug. ^a^Sodium valproate is absolutely contraindicated in women of childbearing potential. ^b^CGRP monoclonal antibodies target CGRP or its receptor.

In this Consensus Statement, we provide a ten-step approach to the diagnosis and management of migraine (Fig. 1). Development of this approach was initiated by the Danish Headache Society, and the Consensus Statement is endorsed by the European Headache Federation (EHF) and the European Academy of Neurology (EAN). The aim of the approach is to support care and clinical decision-making by primary care practitioners, neurologists and headache specialists alike.

## Methods

The Danish Headache Society and its representatives (A.K.E., H.A., H.W.S. and M.Ashina) conceived a European Consensus Statement on the diagnosis and clinical management of migraine. A formal proposal, including a suggested list of authors, was prepared and submitted to the Board of Directors of the EHF, the Chairs of the EAN Headache Panel and the Chair of the EAN Scientific Committee. The proposal was approved by unanimous decision and a European expert panel was convened to develop this Consensus Statement. Three authors (H.A., T.J.S. and M.Ashina) identified the ten most important steps in diagnosis and management of migraine through email correspondence. Once these steps were agreed, seven authors (A.K.E., H.A., S.K., H.-C.D., H.W.S., T.J.S. and M.Ashina) wrote the initial draft.

For each of the ten steps, a structured literature search was performed in April 2021 using the PubMed database. We searched for “migraine” in combination with the terms “diagnosis”, “treatment”, “therapies”, “treatment outcome” or “prognosis”. We excluded publications written in a language other than English. We also selected additional articles deemed relevant from a search of the reference lists of the originally identified articles. The content was targeted towards a broad readership of primary care practitioners, neurologists and headache specialists.

In continuous email correspondence, all authors reviewed the initial draft and contributed to all subsequent drafts. Whenever possible, recommendations were based on interpretation of findings from systematic reviews and meta-analyses, relying on expert opinion only when scientific evidence was limited or unavailable. The views of each author were taken fully into consideration and revisions were made until unanimous consensus was reached. Four rounds of review were required to establish consensus.

## Step 1: When to suspect migraine

In the third edition of the International Classification of Headache Disorders (ICHD-3), migraine is classified into three main types^4^: migraine without aura, migraine with aura, and chronic migraine. The clinical characteristics of each must be considered to ensure an accurate diagnosis.

### Migraine without aura

Migraine without aura is characterized by recurrent headache attacks that last 4–72 h^4^. Typical features of an attack include a unilateral location, pulsating quality, moderate or severe pain intensity, and aggravation by routine physical activity^4,13^. However, bilateral pain is not uncommon; population-based data indicate that ~40% of individuals with migraine report bilateral pain during attacks^5^. The most common associated symptoms are photophobia, phonophobia, nausea and vomiting^4,13^. Before the onset of pain, prodromal symptoms can include a depressed mood, yawning, fatigue and cravings for specific foods^14^. After resolution of the headache, postdromal symptoms can last up to 48 h and often include tiredness, concentration difficulties and neck stiffness^15^.

### Migraine with aura

Approximately one third of individuals with migraine experience aura^5^, either with every attack or with some attacks. Aura is defined as transient focal neurological symptoms that usually precede, but sometimes accompany, the headache phase of a migraine attack^4^. In >90% of affected individuals, aura manifests visually^4,16^, classically as fortification spectra^4^. Sensory symptoms occur in ~31% of affected individuals and are usually experienced as predominantly unilateral paraesthesia (pins and needles and/or numbness) that spreads gradually in the face or arm^16^.

Less common aura symptoms include aphasic speech disturbance, brainstem symptoms (such as dysarthria and vertigo), motor weakness (in hemiplegic migraine) and retinal symptoms (for example, repeated monocular visual disturbances)^4^. Aura symptoms can be similar to those of transient ischaemic attacks (TIA), but can be differentiated on the basis that aura symptoms often spread gradually (over ≥5 min) and occur in succession, whereas symptoms of a TIA have a sudden, simultaneous onset^4^.

Notably, migraine with aura and migraine without aura can coexist. Many individuals with migraine with aura also experience attacks that are not preceded by aura^4^. In such cases, migraine with aura and migraine without aura should both be diagnosed.

### Chronic migraine

Chronic migraine is defined as ≥15 headache days per month for >3 months and fulfilment of ICHD-3 criteria for migraine on ≥8 days per month^4^. Chronic migraine is not a static entity and reversion to episodic migraine is not unusual. Similarly, retransformation to chronic migraine can subsequently occur^17^.

### Family history of migraine

Migraine has a strong genetic component and its prevalence is higher among people with directly affected first-degree relatives than among the general population^18,19^. Family history is, therefore, an important part of the medical history and is often positive in patients with migraine, although it might be under-reported by patients^20^.

### Recommendations

- Suspect migraine without aura in a person with recurrent moderate to severe headache, particularly if pain is unilateral and/or pulsating, and when the person has accompanying symptoms such as photophobia, phonophobia, nausea and/or vomiting.
- Suspect migraine with aura in a person with the symptoms above and recurrent, short-lasting visual and/or hemisensory disturbances.
- Suspect chronic migraine in a person with ≥15 headache days per month.
- Suspicion of migraine should be strengthened by a family history of migraine and if onset of symptoms is at or around puberty.

## Step 2: Diagnosis of migraine

The medical history is the mainstay of migraine diagnosis; with the assistance of a range of published aids (see the section Diagnostic aids), a full history should enable systematic application of the criteria set out in the ICHD-3. Physical examination is most often confirmatory and further investigations (for example, neuroimaging, blood samples or lumbar puncture) are occasionally required to confirm or reject suspicions of secondary causes for headache.

### Medical history

An adequate medical history must include at least the following: age at onset of headache; duration of headache episodes; frequency of headache episodes; pain characteristics (for example, location, quality, severity, aggravating factors and relieving factors); accompanying symptoms (for example, photophobia, phonophobia, nausea and vomiting); aura symptoms (if any); and history of acute and preventive medication use. All are essential for the application of the ICHD-3 criteria.

### Diagnostic criteria

The ICHD-3 criteria^4^ (Box 1), which were developed by the International Headache Society, set out the clinical features that establish the diagnosis of migraine and its types and subtypes. These criteria prioritize specificity over sensitivity, so an additional set of criteria are given for a diagnosis of probable migraine, which is defined as “migraine-like attacks missing one of the features required to fulfil all criteria for a type or subtype of migraine”^4^. Probable migraine is a diagnosis pending confirmation during early follow-up.

Box 1 ICHD-3 diagnostic criteria for primary headache disorders^4^ **Migraine without aura**

1. At least five attacks that fulfil criteria 2–5
2. Headache attacks that last 4–72 h when untreated or unsuccessfully treated
3. Headache has at least two of the following four characteristics:
  unilateral location
  pulsating quality
  moderate or severe pain intensity
  aggravation by, or causing avoidance of, routine physical activity (for example, walking or climbing stairs)

4. At least one of the following during the headache:
  nausea and/or vomiting
  photophobia and phonophobia
5. Not better accounted for by another ICHD-3 diagnosis

**Migraine with aura**

1. At least two attacks that fulfil criteria 2 and 3
2. One or more of the following fully reversible aura symptoms:
  visual
  sensory
  speech and/or language
  motor
  brainstem
  retinal

3. At least three of the following six characteristics:
  at least one aura symptom spreads gradually over ≥5 min
  two or more aura symptoms occur in succession
  each individual aura symptom lasts 5–60 min
  at least one aura symptom is unilateral
  at least one aura symptom is positive
  the aura is accompanied with or followed by headache within 60 min
4. Not better accounted for by another ICHD-3 diagnosis

**Chronic migraine**

1. Headache (migraine-like or tension-type-like) on ≥15 days/month for >3 months that fulfil criteria 2 and 3
2. Attacks occur in an individual who has had at least five attacks that fulfil the criteria for migraine without aura and/or for migraine with aura
3. On ≥8 days/month for >3 months, any of the following criteria are met:
  criteria 3 and 4 for migraine without aura
  criteria 2 and 3 for migraine with aura
  believed by the patient to be migraine at onset and relieved by a triptan or ergot derivative

4. Not better accounted for by another ICHD-3 diagnosis

**Medication-overuse headache**

1. Headache on ≥15 days/month in an individual with a pre-existing headache disorder
2. Regular overuse for >3 months of one or more drugs that can be taken for acute and/or symptomatic treatment of headache (regular intake of one or more non-opioid analgesics on ≥15 days/month for ≥3 months or any other acute medication or combination of medications on ≥10 days/month for ≥3 months)
3. Not better accounted for by another ICHD-3 diagnosis

### Diagnostic aids

Headache diaries are useful diagnostic aids that can also be used to re-evaluate the diagnosis whenever needed (Box 2). Daily diary entries record information on the pattern and frequency of headaches and its accompanying symptoms (for example, nausea, photophobia and phonophobia), as well as use of acute medications (Box 2). Diaries should not be conflated with headache calendars, which typically include less information but are useful in the follow-up assessment of patients. Headache calendars should be used to record, at minimum, the frequency of migraine, the frequency and intensity of headaches, and headache-related events, such as acute and preventive medication use and menstruation (Box 2).

The emergence and refinement of electronic headache diaries and calendars are important developments, as these are likely to facilitate acquisition of more detailed information without markedly compromising compliance. Compliance with headache diaries can be an issue, particularly in primary care; for example, in one population-based study of patients who reported frequent headaches, only 46% of participants completed the study^21^.

Diagnosis of migraine can also be facilitated by use of screening instruments that evaluate whether a patient’s clinical features suggest migraine (Box 2). After use of such screening instruments, diagnosis should be confirmed by a review of the medical history and/or use of a diagnostic headache diary. Validated screening instruments include the three-item ID-Migraine questionnaire^22^ and the five-item Migraine Screen Questionnaire (MS-Q)^23^. The ID-Migraine questionnaire has a sensitivity of 0.81, a specificity of 0.75 and a positive predictive value of 0.93 when compared with ICHD-based diagnosis by a headache specialist^22^. The MS-Q instrument has a sensitivity of 0.93, a specificity of 0.81 and a positive predictive value of 0.83 (ref.^23^). Both instruments have been translated and validated for use in several languages^24–27^.

Box 2 Diagnostic aids and screening tools **Headache diary**

Headache diaries are useful diagnostic aids and can also, if needed, assist with re-evaluation of diagnosis at follow-ups (see Related links for an example headache diary).

**Headache calendar**

Headache calendars are useful in follow-ups for recording the temporal occurrence of headaches and related events, such as menstruation (see Related links for an example headache calendar).

**Three-item Identify Migraine questionnaire**

The three-item Identify Migraine (ID-Migraine) questionnaire identifies individuals who are likely to have migraine on the basis of their answers to three questions regarding headache-associated nausea, photophobia and disability^22^.

**Migraine Screen Questionnaire**

The Migraine Screen Questionnaire (MS-Q), like ID-Migraine, is designed to screen patients for migraine but includes five questions regarding headache frequency, intensity and length, headache associated nausea, photophobia and phonophobia, and disability^23^.

### Differential diagnoses

Differential diagnoses for migraine include other primary headache disorders (Table 1) and some secondary headache disorders (Table 2). Distinction from other primary headache disorders is a prerequisite for successful management, whereas distinction from secondary headache disorders is crucial because some of these disorders are serious and potentially life-threatening (for example, meningitis and subarachnoid haemorrhage) (Table 2).

**Table 1:** Characteristics of primary headache disorders

Headache disorder	Headache duration	Headache location	Pain intensity	Pain characteristics	Accompanying symptoms	Routine physical activity
Migraine	4–72 h	Usually unilateral	Usually moderate or severe	Usually pulsating	Photophobia, phonophobia, nausea, vomiting	Often aggravated by routine physical activity
Tension-type headache	Hours to days or unremitting	Usually bilateral or circumferential	Usually mild or moderate	Usually pressing or tightening	Often none; sometimes photophobia or phonophobia (but not both); sometimes mild nausea in chronic tension-type headache	Not aggravated by routine physical activity
Cluster headache	15–180 min	Strictly unilateral and orbital, supraorbital, and/or temporal	Severe or very severe	Overwhelming	Ipsilateral to the headache: cranial autonomic symptoms, such as conjunctival injection, lacrimation, and nasal congestion	Restlessness or agitation

**Table 2:** Red flags associated with secondary headaches^31,32^

When to look	Red flag	Indication
Patient history	Thunderclap headache	Subarachnoid haemorrhage
	Atypical aura	Transient ischaemic attack, stroke, epilepsy, arteriovenous malformations
	Head trauma	Subdural haematoma
	Progressive headache	Intracranial space-occupying lesion
	Headache aggravated by postures or manoeuvres that raise intracranial pressure	Intracranial hypertension or hypotension
	Headache brought on by sneezing, coughing or exercise	Intracranial space-occupying lesion
	Headache associated with weight loss and/or change in memory or personality	Suggests secondary headache
	Headache onset at >50 years of age	Suggests secondary headache; consider temporal arteritis
Physical examination	Unexplained fever	Meningitis
	Neck stiffness	Meningitis, subarachnoid haemorrhage
	Focal neurological symptoms	Suggests secondary headache
	Weight loss	Suggests secondary headache
	Impaired memory and/or altered consciousness or personality	Suggests secondary headache

Tension-type headache (TTH) is the only other paroxysmal headache disorder that is prevalent in the general population^28^. TTH lacks the symptoms that accompany migraine and usually involves bilateral, mild to moderate pain with a pressing or tightening quality that is not aggravated by routine physical activity^4,28^ (Table 1).

Cluster headache is a much less prevalent primary headache disorder that affects ~0.1% of the general population^29^. Its features are highly characteristic and include frequently recurrent but short-lasting attacks (15–180 min) of strictly unilateral headache of severe or very severe intensity^4^. The head pain is accompanied by ipsilateral cranial autonomic symptoms, such as conjunctival injection, lacrimation and nasal congestion^4^ (Table 1).

Medication-overuse headache (MOH) is a secondary headache disorder that is an important differential diagnosis for chronic migraine^30^ (Box 1). This disorder commonly develops from overuse of acute medication to treat migraine attacks, so the two disorders are often conflated (see Step 8 for more on MOH).

Some other secondary headache disorders can present with features that suggest migraine, but specific red flags should create suspicion (Table 2). Red flags in the medical history include thunderclap headache, atypical aura and head trauma. Red flags in the physical examination include unexplained fever, impaired memory and focal neurological symptoms (Table 2). These red flags are indications for further investigation, such as neuroimaging, blood samples or lumbar puncture^31^.

### Need for neuroimaging

The only role for neuroimaging in the diagnosis of headache is to confirm or exclude causes of secondary headache that are suspected on the basis of red flags in the medical history and/or physical examination^32,33^. Otherwise, neuroimaging is not only rarely necessary in the diagnostic work-up of migraine but can be harmful, as it can involve exposure to ionizing radiation^33,34^. When needed for investigation of possible secondary headache disorders, MRI is preferred to CT, as it offers a higher resolution and does not involve exposure to ionizing radiation^35,36^. However, MRI can reveal clinically insignificant abnormalities (for example, white matter lesions, arachnoid cysts and meningiomas), which can alarm the patient and lead to further unnecessary testing^33,37,38^.

### Recommendations

- Take a careful medical history, applying the ICHD-3 criteria.
- Use validated diagnostic aids and screening tools, such as headache diaries, the three-item ID-Migraine questionnaire and the five-item Migraine Screen Questionnaire.
- Consider differential diagnoses, including other primary headache disorders and secondary headache disorders.
- Use neuroimaging only when a secondary headache disorder is suspected.

## Step 3: Education and patient centricity

Patient centricity and education have important roles in the management of migraine. Indeed, optimal outcomes are unlikely when these aspects are not given sufficient attention.

### Explanation, reassurance and objectives

Patient satisfaction is a key management outcome and treatment success depends on it but most people with migraine report at least one perceived unmet treatment need^39^. Unrealistic expectations constitute a major obstacle to achieving patient satisfaction — a common misconception among patients is that effective treatment means cure of their migraine^32,40^. Clinicians must therefore disabuse patients of this belief without being overly negative. A realistic objective is a return of control from the disease to the patient with treatment that mitigates attack-related disability (by reducing attack frequency, attack duration and/or pain intensity) to an extent that the patient can continue with life with as little hindrance as possible.

Non-adherence is also an obstacle to effective treatment^41^ and requires management. Education is the solution — clinicians must explain to the patient both the disease and the principles of managing it effectively, including instruction on the correct use of medication, potential adverse effects and what to do about them, and the importance of avoiding medication overuse. Such education can require time that is not available, but freely available patient information leaflets can support patient education^32^.

### Predisposing factors and triggers

Contrary to popular belief, predisposing and trigger factors are of limited importance in migraine, and their role is often overemphasized^42^. An important exception is menstruation, as some women’s migraine attacks are exclusively or frequently menstruation-related. True trigger factors are often self-evident. Moreover, aggravating factors should not be conflated with predisposing factors. The former worsen headache during migraine attacks (for example, physical activity), whereas predisposing factors increase susceptibility to the development of a migraine attack (for example, poor sleep quality, poor physical fitness or stress).

Nevertheless, if predisposing and trigger factors can be correctly identified and subsequently avoided (which is often not possible), some headache control might be achievable without further intervention^43^. For instance, lifestyle changes can benefit patients with poor sleep quality or physical fitness, though any changes should not result in unnecessary avoidance behaviour, which can itself damage quality of life.

### Individualized therapy

Multiple effective acute and preventive therapies are available for migraine. When selecting from these therapies, the objective is that each patient receives the therapy that provides the best personal outcome. Unfortunately, no a priori basis for selection currently exists, at least for acute therapy. Optimal individualized therapy is therefore currently best achieved with a stepped care approach, set out in detail in Step 4.

### Recommendations

- Provide every patient with a full explanation of migraine as a disease and of the principles of its management.
- Consider predisposing and trigger factors, but keep in mind that true trigger factors are often self-evident.
- Adhere to the principles of stepped care to achieve optimal individualized therapy (see Step 4).

## Step 4: Acute treatment

**Fig. 2:**
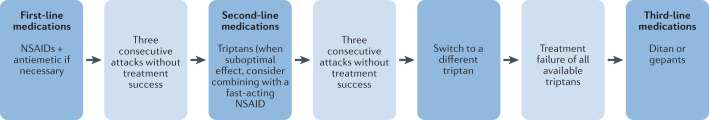
Stepped care across migraine attacks. Preventive therapy, in addition, may be indicated at any stage. In general, initiation of preventive therapy is indicated in patients who are adversely affected on ≥2 days per month despite acute treatment optimized according to the stepped care approach. NSAID, non-steroidal anti-inflammatory drug.

Acute treatments can be classified as first-line, second-line, third-line and adjunct (Table 3), and should be used in a stepped care approach^32^ (Fig. 2). Our recommendations for each line of treatment are outlined below. The medications at each stage were selected on the basis of efficacy, tolerability, safety, cost and availability.

**Table 3:** Acute migraine treatment

Drug class	Drug	Dosage and route	Contraindications
***First-line medication***			
NSAIDs	Acetylsalicylic acid	900–1,000 mg oral	Gastrointestinal bleeding, heart failure
	Ibuprofen	400–600 mg oral	
	Diclofenac potassium	50 mg oral (soluble)	
Other simple analgesics (if NSAIDs are contraindicated)	Paracetamol	1,000 mg oral	Hepatic disease, renal failure
Antiemetics (when necessary)	Domperidone	10 mg oral or suppository	Gastrointestinal bleeding, epilepsy, renal failure, cardiac arrhythmia
	Metoclopramide	10 mg oral	Parkinson disease, epilepsy, mechanical ileus
***Second-line medication***			
Triptans	Sumatriptan	50 or 100 mg oral or 6 mg subcutaneous or 10 or 20 mg intranasal	Cardiovascular or cerebrovascular disease, uncontrolled hypertension, hemiplegic migraine, migraine with brainstem aura
	Zolmitriptan	2.5 or 5 mg oral or 5 mg intranasal	
	Almotriptan	12.5 mg oral	
	Eletriptan	20, 40 or 80 mg oral	
	Frovatriptan	2.5 mg oral	
	Naratriptan	2.5 mg oral	
	Rizatriptan	10 mg oral tablet (5 mg if treated with propranolol) or 10 mg mouth-dispersible wafers	
***Third-line medication***			
Gepants	Ubrogepant	50, 100 mg oral	Co-administration with strong CYP3A4 inhibitors
	Rimegepant	75 mg oral	Hypersensitivity, hepatic impairment
Ditans	Lasmiditan	50, 100 or 200 mg oral	Pregnancy, concomitant use with drugs that are P-glycoprotein substrates

### First-line medication

Over-the-counter analgesics are used worldwide for acute migraine treatment^44^. Those with proven efficacy include non-steroidal anti-inflammatory drugs (NSAIDs), and the strongest evidence supports use of acetylsalicylic acid, ibuprofen and diclofenac potassium as first-line medications^45–47^. Paracetamol has less efficacy^48^ and should be used only in those who are intolerant of NSAIDs.

### Second-line medication

Patients for whom over-the-counter analgesics provide inadequate headache relief should be offered a triptan. All triptans have well-documented effectiveness, but availability of and access to each vary between countries. Triptans are most effective when taken early in an attack, when the headache is still mild^49,50^. However, no evidence supports the use of triptans during the aura phase of a migraine attack. If one triptan is ineffective, others might still provide relief^51,52^. When all other triptans have failed or in patients who rapidly reach peak headache intensity or cannot take oral triptans because of vomiting, sumatriptan by subcutaneous injection can be useful^53^.

Some patients can experience relapses, which are defined as a return of symptoms within 48 h after apparently successful treatment. Upon relapse, patients can repeat their triptan treatment or combine the triptan with simultaneous intake of fast-acting formulations of naproxen sodium, ibuprofen lysine or diclofenac potassium^54,55^. However, patients should be informed that repeating the treatment does not preclude further relapses and ultimately increases the risk of developing MOH.

### Third-line medication

If all available triptans fail after an adequate trial period (no or insufficient therapeutic response in at least three consecutive attacks) or their use is contraindicated, alternatives are currently limited. Ditans or gepants could be used, but their availability is currently very limited. Lasmiditan is the only ditan approved for acute treatment of migraine, and ubrogepant and rimegepant are the only gepants approved. Indirect comparison of data from randomized controlled trials suggests that the efficacy of lasmiditan is comparable to that of triptans^56–58^, but its use is associated with temporary driving impairment, which is likely to discourage widespread use. Individuals who take lasmiditan might be unable to self-assess their driving competence and should not operate machinery for at least 8 h after intake.

### Adjunct medication

For patients who experience nausea and/or vomiting during migraine attacks, prokinetic antiemetics such as domperidone and metoclopramide are useful oral adjuncts.

### Medications to avoid

Oral ergot alkaloids are poorly effective and potentially toxic, and should not be used as a substitute for triptans^59^. The efficacy of opioids and barbiturates is questionable, and both are associated with considerable adverse effects and the risk of dependency^60^. All of these medications should, therefore, be avoided for the acute treatment of migraine.

### Recommendations

- Offer acute medication to everyone who experiences migraine attacks.
- Advise use of acute medications early in the headache phase of the attack, as effectiveness depends on timely use with the correct dose.
- Advise patients that frequent, repeated use of acute medication risks development of MOH.
- Use NSAIDs (acetylsalicylic acid, ibuprofen or diclofenac potassium) as first-line medication.
- Use triptans as second-line medication.
- Consider combining triptans with fast-acting NSAIDs to avert recurrent relapse.

- Consider ditans and gepants as third-line medications.
- Use prokinetic antiemetics (domperidone or metoclopramide) as adjunct oral medications for nausea and/or vomiting.
- Avoid oral ergot alkaloids, opioids and barbiturates.

## Step 5: Preventive treatment

### Initiation and termination

In patients whose migraine continues to impair their quality of life despite optimized acute therapy, additional preventive therapy should be considered (Table 4). In practice, patients who are considered for preventive treatment remain adversely affected on at least 2 days per month^32^, although this should not be regarded as an absolute rule^32^. Aside from migraine frequency, clinicians should always consider factors such as the severity of attacks, the duration of attacks (for example, menstruation-related attacks tend to last longer) and migraine-related disability. A further indication for preventive therapy is overuse of acute medication.

**Table 4:** Preventive migraine treatment

Drug class	Drug	Dosage and route	Contraindications
***First-line medication***			
Beta blockers	Atenolol	25–100 mg oral twice daily	Asthma, cardiac failure, Raynaud disease, atrioventricular block, depression
	Bisoprolol	5–10 mg oral once daily	
	Metoprolol	50–100 mg oral twice daily or 200 mg modified-release oral once daily	
	Propranolol	80–160 mg oral once or twice daily in long-acting formulations	
Angiotensin II-receptor blocker	Candesartan	16–32 mg oral per day	Co-administration of aliskiren
Anticonvulsant	Topiramate	50–100 mg oral daily	Nephrolithiasis, pregnancy, lactation, glaucoma
***Second-line medication***			
Tricyclic antidepressant	Amitriptyline	10–100 mg oral at night	Age <6 years, heart failure, co-administration with monoamine oxidase inhibitors and SSRIs, glaucoma
Calcium antagonist	Flunarizine	5–10 mg oral once daily	Parkinsonism, depression
Anticonvulsant	Sodium valproate^a^	600–1,500 mg oral once daily	Liver disease, thrombocytopenia, female and of childbearing potential
***Third-line medication***			
Botulinum toxin	OnabotulinumtoxinA	155–195 units to 31–39 sites every 12 weeks	Infection at injection site
Calcitonin gene-related peptide monoclonal antibodies	Erenumab	70 or 140 mg subcutaneous once monthly	Hypersensitivity Not recommended in patients with a history of stroke, subarachnoid haemorrhage, coronary heart disease, inflammatory bowel disease, chronic obstructive pulmonary disease or impaired wound healing
	Fremanezumab	225 mg subcutaneous once monthly or 675 mg subcutaneous once quarterly	
	Galcanezumab	240 mg subcutaneous, then 120 mg subcutaneous once monthly	
	Eptinezumab	100 or 300 mg intravenous quarterly	

SSRI, selective serotonin reuptake inhibitor. ^a^Sodium valproate is absolutely contraindicated in women of childbearing potential.

Efficacy of preventive therapy is rarely observed immediately. Only after several weeks or months can efficacy be ascertained, so patients should be discouraged from abandoning the treatment in these early stages on the grounds of apparent inefficacy^32^. If a therapeutic dose of an oral preventive medication is ineffective after 2–3 months, an alternative should be tried^32,61,62^. For monoclonal antibody treatments that target calcitonin gene-related peptide (CGRP) or its receptor, efficacy should be assessed only after 3–6 months. For onabotulinumtoxinA, efficacy should be assessed after 6–9 months.

Failure of one preventive treatment does not predict failure of treatment with other drug classes, except when failure is due to poor adherence. Treatment adherence is often very poor but can be improved by simplified dosing schedules (once daily or less)^32^. For most preventive medications, clinical experience suggests that pausing can be considered when treatment has been successful for 6–12 months^32^. The purpose of pausing is to ascertain whether preventive treatment can be stopped, which minimizes the risk of unnecessary drug exposure and allows some patients to manage their migraine with acute medications only. A useful measure to quantify the degree of preventive treatment success is to calculate the percentage reduction in monthly migraine days or monthly headache days of moderate-to-severe intensity. However, a pragmatic approach is needed and clinicians should decide to pause preventive therapy on a case-by-case basis.

### Current standard of care

As for acute medications, preventive treatments can be classified as first-line, second-line and third-line options (Table 4). However, choice of medication and the order of use depend on local practice guidelines and local availability, costs and reimbursement policies.

First-line medications are beta blockers without intrinsic sympathomimetic activity (atenolol, bisoprolol, metoprolol or propranolol)^63^, topiramate^64^ and candesartan^65,66^. If these fail, second-line medications include flunarizine^67^, amitriptyline^68^ and sodium valproate^69^, although valproate is strictly contraindicated in women of childbearing potential, which greatly limits its utility in migraine^70–72^. Third-line medications are the four CGRP monoclonal antibodies erenumab, fremanezumab, galcanezumab and eptinezumab. These antibodies have been approved for the preventive treatment of migraine in the past few years^61^. In Europe, regulatory restrictions limit their use to patients in whom other preventive drugs have failed or are contraindicated^61^.

### Non-pharmacological therapies

A range of non-pharmacological preventive therapies can be used either as adjuncts to acute and preventive medications or instead of them if medication use is contraindicated. Some evidence supports the use of non-invasive neuromodulatory devices^73^, biobehavioural therapy^74^ and acupuncture^75^, although a study of acupuncture indicated that it is not superior to sham acupuncture^76^. Contrary to popular belief, little to no evidence exists for physical therapy^77^, spinal manipulation and dietary approaches^78^. We make no recommendations about other therapeutic options, such as melatonin, magnesium and riboflavin, as limited evidence for their efficacy is available and their use in clinical practice is limited.

### Recommendations

- Consider preventive treatment in patients who are adversely affected by migraine on ≥2 days per month despite optimized acute treatment.
- Use beta blockers (atenolol, bisoprolol, metoprolol or propranolol), topiramate or candesartan as first-line medications.
- Use flunarizine, amitriptyline or (in men) sodium valproate as second-line medications.
- Consider CGRP monoclonal antibodies as third-line medications.
- Consider neuromodulatory devices, biobehavioural therapy and acupuncture as adjuncts to acute and preventive medication or as stand-alone preventive treatment when medication is contraindicated.

## Step 6: Managing migraine in special populations

### Older people

Migraine often remits with older age whereas the incidence of many secondary headaches increases^79–81^. Onset of apparent migraine after the age of 50 years should, therefore, arouse suspicion of an underlying cause. In individuals whose migraine persists from earlier life into later years, clinical management often remains unchanged in practice. Little formal evidence is available with respect to therapeutic approaches in older people with migraine.

Nonetheless, known and possible unknown comorbidities need to be considered, as well as harm that might be caused by drug-specific adverse effects^82^, to which older people are generally more susceptible. For instance, use of triptans in older people is often advised against owing to the relatively high likelihood that these patients have cardiovascular disease and/or cardiovascular risk factors. However, no robust evidence supports an increased risk of cerebrovascular or cardiovascular events in older people owing to triptan use per se^83^. Nonetheless, clinicians are advised to regularly monitor blood pressure in older patients with migraine who use triptans, in addition to periodical assessment of cardiovascular risk factors^84^.

### Children and adolescents

Migraine is common among children and its prevalence increases in adolescence^85^. As in adults, diagnosis is primarily based on the medical history, although the criteria are slightly different — the duration of migraine attacks can be 2 to 72 h^4^. The clinical features of migraine in children and adolescents also differ somewhat from those in adults — the attacks are often shorter^4^, the headache is more often bilateral and less often pulsating, and gastrointestinal disturbances are commonly prominent^32^. Descriptions of these features might be more reliably provided by parents than children, and parents will also provide a better account of lifestyle factors that might need to be addressed^86^.

In children and young adolescents, clinical management usually requires active help from family members and teachers^86^, so education of both is necessary. Bed-rest alone might suffice in children with attacks that have a short duration. When needed, ibuprofen is recommended as first-line medication, at a dose appropriate for body weight^32^. Domperidone can be used for nausea in adolescents aged 12–17 years^87^, although oral administration is unlikely to prevent vomiting.

The evidence base for medication therapy in children and adolescents is confounded by a high placebo response in clinical trials^88,89^. As a consequence, the apparent therapeutic gain is low, and this effect probably explains why a benefit of triptans has not been demonstrated in children. For adolescents aged 12–17 years, multiple NSAIDs and triptans have been approved for acute treatment of migraine^90,91^, and some evidence indicates that nasal spray formulations of sumatriptan and zolmitriptan are the most effective^92^. If acute medication provides insufficient pain relief, referral to specialist care is indicated^32^. In practice, propranolol, amitriptyline and topiramate are used for preventive treatment, although their effectiveness in children and adolescents has not been proven in clinical trials^88,89^.

### Pregnant and breastfeeding women

Migraine often remits during pregnancy, but if treatment is continued, the potential for harm to the fetus demands special consideration^93^. Despite relatively poor efficacy, paracetamol should be used as the first-line medication for acute treatment of migraine in pregnancy^48^; NSAIDs can be used only during the second trimester^93,94^. Triptans should be used only under the strict supervision of a specialist, as the safety data available are limited and originate from post-marketing surveillance; most data relate to the use of sumatriptan^32^. For nausea associated with migraine in pregnancy, metoclopramide can be used^94,95^.

Preventive migraine medications are best avoided during pregnancy owing to the potential for fetal harm. However, if preventive therapy is considered clinically indicated because of frequent and disabling migraine attacks, the best available safety data support the use of propranolol or, if propranolol is contraindicated, amitriptyline. Both should be used under specialist supervision to adequately monitor any potential fetal harm^32^. Topiramate, candesartan and sodium valproate are contraindicated; sodium valproate is known to be teratogenic, so must not be used^70,94^, and the use of topiramate and candesartan is associated with adverse effects on the fetus.

Migraine medication therapy in the post-partum period also requires caution because of potential risks to the infant. Paracetamol is the preferred acute medication, although ibuprofen and sumatriptan are also considered safe^94^. If preventive medication is required, propranolol is the recommended first choice as it has the best safety profile^94^. Pharmacological treatments for migraine during pregnancy and breastfeeding have been reviewed in more detail elsewhere^94^.

### Women with menstrual migraine

Approximately 8% of women with migraine experience migraine attacks that are exclusively related to their menstruation, referred to as pure menstrual migraine^96,97^. If optimized acute medication therapy does not suffice for these patients, initiation of perimenstrual preventive treatment should be considered. This approach typically involves daily intake of a long-acting NSAID (for example, naproxen) or triptan (for example, frovatriptan or naratriptan) for 5 days, beginning 2 days before the expected first day of menstruation^98–101^. Some women with pure menstrual migraine without aura benefit from continuous use (that is, without a break) of combined hormonal contraceptives. By contrast, combined hormonal contraceptives are contraindicated in women with migraine with aura regardless of any association with their menstrual cycle, owing to an associated increase in the risk of stroke^32^.

### Recommendations

- In patients with apparent late-onset migraine, suspect an underlying cause.
- In older people, consider the higher risks of secondary headache, comorbidities and adverse events with older age.
- In children and adolescents with migraine, bed rest alone might suffice; if not, use ibuprofen for acute treatment and propranolol, amitriptyline or topiramate for prevention.
- In pregnant or breastfeeding women, use paracetamol for acute treatment and avoid preventive medication whenever possible.
- In women with menstrual migraine, consider perimenstrual preventive therapy with a long-acting NSAID or triptan.

## Step 7: Follow-up, treatment response and failure

Active follow-up is the only appropriate means of determining outcome and provides the opportunity to review both diagnosis and treatment strategies. The response to treatment should be evaluated within 2–3 months after initiation or a change in treatment, and regularly thereafter, though not necessarily at short intervals (for example, 6–12 months). Evaluation of treatment responses should include a review of effectiveness, adverse events and adherence.

Key outcome measures for effectiveness are attack frequency, attack severity and migraine-related disability^32^. Attack frequency is usually measured in headache or migraine days per month. Severity is usually expressed as pain intensity rather than functional consequence, which should be separately assessed. Headache calendars are extremely useful for capturing these measures and require little time commitment if completed only on symptomatic days^32^. In addition, headache calendars are valuable for monitoring acute medication use. At follow-up assessments, the self-administered Migraine Treatment Optimization Questionnaire (mTOQ-4) can be used to evaluate the effectiveness of acute medications^102^, whereas the self-completed eight-item HURT questionnaire (Headache Under-Response to Treatment) can be used to assess the effectiveness of an intervention and generates suggestions for changes to improve effectiveness^103^ (Box 3).

Box 3 Tools for evaluation of treatment response **HURT questionnaire**

The Headache Under-Response to Treatment (HURT) questionnaire is an eight-item, self-administered questionnaire developed specifically to guide follow-up in primary care^103^. The questionnaire assesses treatment outcome in several domains, and responses are coupled to suggested changes in management. It has been validated for clinical use in English and Arabic^133,134^ and is available online in 12 languages (see Related links for where to access the HURT questionnaire).

**mTOQ-4**

The Migraine Treatment Optimization Questionnaire (mTOQ-4) is a self-administered questionnaire that can be used to assess acute treatment, including treatment efficacy^102^. This questionnaire has been validated for use in primary care and used in several studies to assess treatment outcomes^102,118,135^.

### When treatment fails

A conclusion that treatment has failed should be made with caution and must always be preceded by a thorough review of the underlying reasons. In some cases, apparent failures might be remediable, such as when failure is due to poor adherence or suboptimal dosing^32^. Whereas some patients benefit from higher doses, others might benefit from lower doses that have fewer adverse effects and therefore improve adherence. Alternatives when first-line medications fail are outlined above (see Step 4 and Step 5). If all treatments fail, the diagnosis should be questioned and specialist referral is indicated^32^.

### When specialist referral is needed

Approximately 90% of people who seek professional care for migraine should be treated in primary care^104^. Referral to specialist care should be reserved for the minority of patients whose condition is diagnostically challenging, difficult to treat or complicated by comorbidities^32^. Specialist care provides access to greater expertise maintained by experience and to multidisciplinary care. However, specialist capacity is limited and the cost is much higher^105^.

### Recommendations

- Evaluate treatment responses shortly after initiation (after 2–3 months) or a change of treatment and regularly thereafter (every 6–12 months).
- Evaluate the effectiveness of treatment by assessing attack frequency, attack severity and migraine-related disability.
- When outcomes are suboptimal, review the diagnosis, treatment strategy, dosing and adherence.
- If all treatment fails, question the diagnosis and consider specialist referral.

## Step 8: Managing complications

### Medication overuse headache

MOH is a chronic headache disorder characterized by headache on ≥15 days per month. It develops over a variable period of time in patients with a pre-existing headache disorder as a result of regular overuse of acute or symptomatic headache medication^4^. Patients with migraine account for approximately two thirds of all cases of MOH, although this estimate is based on limited evidence and might be too low^106^.

Withdrawal of the overused medication is the necessary and only remedy for MOH^107^. Expert consensus is that abrupt withdrawal is preferable to slow withdrawal, except for opioids^30^. This process can be managed in primary care unless addictive drugs, such as opioids, are involved^108,109^. Patient education is a key component of the clinical management of MOH, as withdrawal is usually followed by worsening before recovery^30,110^. Preventive therapy (pharmacological and/or non-pharmacological) appropriate to the antecedent headache can be started in parallel with acute medication withdrawal or upon re-emergence of the headache disorder^30^, although this topic remains a subject of debate^111,112^.

### Transformation to chronic migraine

Some estimates suggest that up to 3% of patients with episodic migraine experience transformation to chronic migraine each year^113^. The reliability of such estimates is uncertain because chronic migraine is often conflated with MOH^114^, but transformation to chronic migraine does occur. Recognized risk factors include female sex, a high headache frequency, inadequate treatment, overuse of acute medications and a range of comorbidities, including depression, anxiety and obesity^115–118^. Recognition of these risk factors is part of good clinical management, as their modification can prevent transformation.

Once chronic migraine has developed, its management is challenging and referral to specialist care is usually necessary^32^. If MOH, which frequently causes symptoms that suggest chronic migraine, can be ruled out, then a preventive treatment should be established^114^. Individuals with chronic migraine should also be educated on the modifiable risk factors for chronic migraine so that they can make lifestyle changes that might help.

Preventive medications for which evidence supports effectiveness in chronic migraine include topiramate^119^, onabotulinumtoxinA^120^ and CGRP monoclonal antibodies^121^. Topiramate is the drug of first choice owing to its much lower cost. Regulatory restrictions generally limit the use of onabotulinumtoxinA and CGRP antibodies to patients in whom two or three other preventive medications have failed, despite the fact that topiramate is the only other treatment with evidence supporting its use. Three CGRP antibodies (erenumab, fremanezumab and galcanezumab) have been proven to be beneficial for patients in whom at least two other preventive medications have failed^122–124^. As in episodic migraine, the choice of preventive medication and their order of use depends on local practice guidelines, availability, cost and reimbursement policies. No robust data from random controlled trials support the use of beta blockers, candesartan or amitriptyline for the preventive treatment of chronic migraine, although they are commonly used in clinical practice.

### Recommendations

- Educate patients with migraine about the risk of MOH with frequent overuse of acute medication.
- Manage established MOH by explanation and withdrawal of the overused medication; abrupt withdrawal is preferred, except for opioids.
- Recognize and, when possible, modify risk factors for the transformation of episodic migraine to chronic migraine.
- Refer patients with chronic migraine to specialist care.
- Once MOH is ruled out, initiate preventive medication therapy for chronic migraine; evidence-based treatment options are topiramate, onabotulinumtoxinA and CGRP monoclonal antibodies.

## Step 9: Recognizing and managing comorbidities

Migraine is associated with anxiety, depression, sleep disturbances and chronic pain conditions (for example, neck and lower back pain)^125–129^. These associations are more pronounced in people with chronic migraine than in those with episodic migraine^130^. Obesity is also an important risk factor for transformation from episodic migraine to chronic migraine and should be accounted for in the clinical evaluation^131^. Furthermore, migraine with aura has been associated with cardiovascular events in women^132^.

Recognition of comorbid conditions in migraine is important because they can influence drug choice. For example, topiramate is the preferred treatment for patients with obesity owing to its association with weight loss. For patients with depression or sleep disturbances, amitriptyline is most likely to be of benefit. Recognition of comorbidities is also important because their alleviation can improve treatment outcomes for migraine, and vice versa.

### Recommendations

- Ensure that comorbidities are identified in patients with migraine, as they can affect treatment choice and outcomes.
- Adjust treatments accordingly and consider possible interactions between drug-related adverse effects and the patient’s comorbidity profile.

## Step 10: Long-term follow-up

Long-term management of migraine should be the responsibility of primary care. Referral from specialist care back to primary care should be timely, coordinated with the general practitioner and accompanied by a comprehensive treatment plan that includes recommendations for re-evaluation and steps to be taken for each of the likely outcomes. In general, timely return to primary care can be made once the patient experiences sustained efficacy with preventive therapy for up to 6 months with no substantial treatment-related adverse effects.

In primary care, the main goal of follow-up is to maintain stability of adequate outcomes, whether achieved in primary or specialist care, and to react appropriately to any change that might call for review. Neither purpose requires regular routine contact, which should, therefore, be avoided unless necessary in the context of repeat prescriptions. Instead, primary care physicians should emphasize patient education and self-efficacy with respect to judging when a return visit is necessary.

### Recommendations

- Primary care should be responsible for the long-term management of patients with migraine, maintaining stability and reacting to change.
- Referral from specialist back to primary care should be timely and accompanied by a comprehensive treatment plan.
- The patient can be referred back to primary care once sustained efficacy with preventive therapy for up to 6 months is obtained with no substantial treatment-related adverse effects.

## Conclusions

Migraine is a ubiquitous neurological disorder that adds substantially to the global burden of disease. Despite the existence of comprehensive diagnostic criteria and a multitude of therapeutic options, diagnosis and clinical management of migraine remain suboptimal worldwide. This Consensus Statement was developed by experts from Europe to provide generally applicable recommendations for the diagnosis and management of migraine and to promote best clinical practices. The recommendations are based on published evidence and expert opinion, and will be updated when new information and treatments emerge.
